# A Quantitative Evaluation of the Conservation Umbrella of Spotted Owl Management Areas in the Sierra Nevada

**DOI:** 10.1371/journal.pone.0123778

**Published:** 2015-04-23

**Authors:** Ryan D. Burnett, L. Jay Roberts

**Affiliations:** Point Blue Conservation Science, Petaluma, California, Unites States of America; Chinese Academy of Sciences, CHINA

## Abstract

Whether by design or default, single species management often serves as an umbrella for species with similar habitat requirements. In recent decades the focus of National Forest management in the Sierra Nevada of California has shifted towards increasing closed canopy mature forest conditions through the protection of areas occupied by the California Spotted Owl (*Strix occidentalis occidentalis*). To evaluate the implications of these habitat changes and the potential umbrella resulting from a system of owl reserves on the broader avian community, we estimated occupancy of birds inside and outside of Spotted Owl Home Range Core Areas in northeastern California. We used point count data in a multi-species hierarchical Bayesian model incorporating the detection history of 81 species over a two-year time period (2005-2006). A small set of vegetation cover and topography covariates were included in the model to account for broad differences in habitat conditions, as well as a term identifying whether or not a site was within a Core Area. Seventeen species had a negative Core Area effect, seven had a positive effect, and the rest were not significant. Estimated species richness was significantly different with 23.1 species per 100 m radius circle outside Core Areas and 21.7 inside Core Areas. The majority of the species negatively associated with Core Areas are tied to early successional and other disturbance-dependent habitats. Conservation and climate vulnerability rankings were mixed. On average we found higher scores (greater risk) for the species positively associated with Core Areas, but a larger number of species with the highest scores were negatively associated with Core Areas. We discuss the implications for managing the Sierra Nevada ecosystem and illustrate the role of monitoring broader suites of species in guiding management of large complex ecosystems.

## Introduction

Management of large and complex forest landscapes tends to be driven by concern for a handful of species. In some cases this happens simply because of legal requirements to manage for listed species. In other cases this is by design, with limited resources available to monitor myriad species, surrogates such as indicator, focal, and umbrella species have been used to guide conservation and management [1–4]. The literature on effective uses and limitations of such approaches is rich [5–9], though there is little empirical evidence in the literature quantifying the efficacy of surrogate guided management on large groups of co-occurring taxa [10]. One of the implicit assumptions for the use of surrogates is that actions intended to benefit the surrogate will provide for the needs of some pre-defined group of other species or ecosystem elements [3,5,9]. An understanding of the species that benefit from, or may be negatively affected by, management for the surrogate can help identify gaps and ensure that management approaches meet the habitat needs of a greater number of species.

On the western slope of the Sierra Nevada of California, National Forest management is heavily influenced by the California Spotted Owl (*Strix occidentalis occidentalis*)—a mature forest associated species. The owl is both a Forest Service Sensitive Species and Management Indicator Species in the Sierra Nevada, and a California Species of Special Concern [11–13]. The plans currently guiding management of National Forests in the Sierra Nevada planning region focus considerable attention on minimizing impacts to this species by establishing a system of small reserves—Spotted Owl Home Range Core Areas (Core Areas)—to protect areas occupied by the owl [14]. Management actions that substantially reduce canopy cover (e.g. below 50%) or otherwise alter habitat suitability for the Spotted Owl are restricted inside Core Areas [11]. The USFS has a stated goal of promoting these “old-growth” conditions on 40% of their land base in the Sierra Nevada [11]. As a result, outside of Core Areas, silvicultural prescriptions retain high canopy cover and all large trees in order to promote dense late seral forest conditions.

Prior to the employment of the current owl management strategy, the composition and structure of mixed-conifer and true-fir forest—the dominant habitat types on the western slope of the range—were undergoing changes as a result of over a century of timber harvest and decades of fire suppression. Timber harvest has resulted in a substantial range-wide reduction in late-seral old growth forest [15–17]. Historically, fire was the primary agent responsible for creating and maintaining habitat diversity and landscape heterogeneity in the Sierra Nevada [18]. Fire return intervals have been lengthened and the area affected annually by wildfire has been dramatically reduced over the past century [19–21]. As a result there has been a range-wide increase in tree densities [15,19, 22–24], and a decrease in shade intolerant plant assemblages [25–28]. Ongoing fire suppression and management approaches intended to protect old-growth closed-canopy forest associated species, such as the Spotted Owl, may continue these trends over the next several decades [11]. If these trends continue, by the middle of the century the existing Core Areas will have higher canopy cover and larger trees, and a greater percentage of mixed-conifer and true-fir forest will resemble the conditions found today within Core Areas.

Understanding the composition of the avian community in the context of Spotted Owl management areas can provide insight into which species’ needs may or may not be met under such a management approach. With the considerable resources devoted to its study, the substantial amount of the landscape being set aside in a system of reserves, and its influence over management of the majority of the remaining landscape we suggest that management for this single species may have profound effects on the rest of the avian community. However, little empirical evidence exists to suggest which species’ habitats are protected within spotted owl reserves, and which may be negatively impacted by this management strategy.

We used a hierarchical Bayesian multi-species occupancy modeling framework to compare the occupancy and richness of 81 landbird species inside and outside of Core Areas on National Forest land across a 96,542 ha area of the northern Sierra Nevada. We then used three established criteria to evaluate the conservation status of these species to determine the importance of Spotted Owl Core Areas to overall avian conservation in the Sierra Nevada. Our objectives were to quantitatively determine the conservation benefits of Spotted Owl protected areas for the avian community and provide an indication of the species and habitat conditions that might be priorities outside of Core Areas if current trends in canopy cover and tree densities continue on National Forest lands.

## Materials and Methods

### Study location

**Fig 1:**
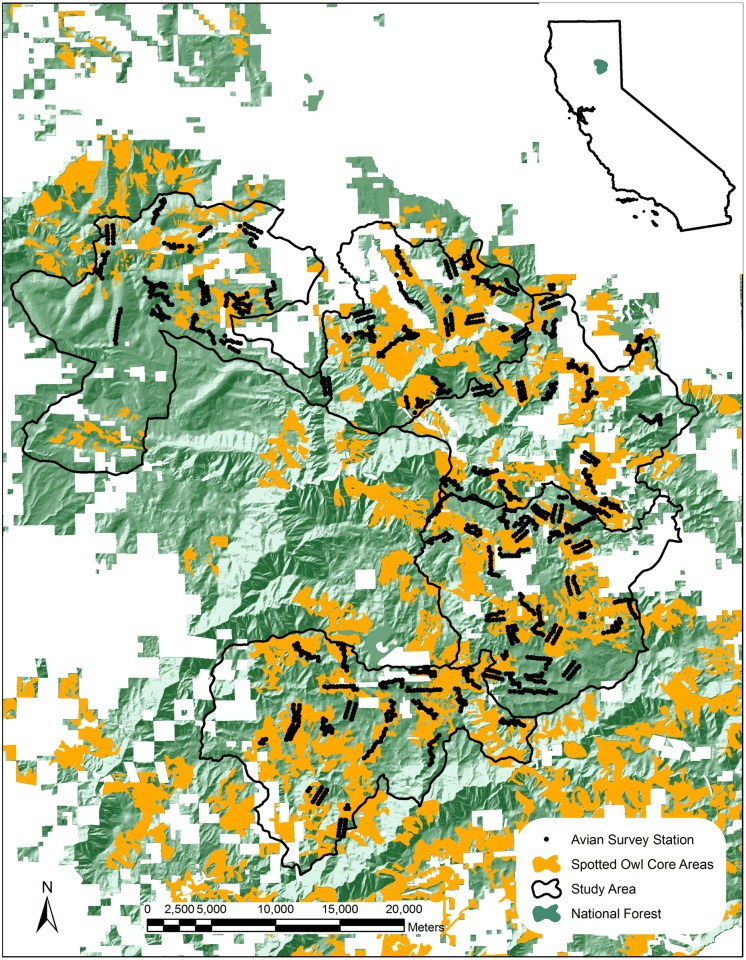
Study location on the Plumas and Lassen National Forests in northeastern California.

Our study occurred within the Plumas and Lassen National Forests at the intersection of the Sierra Nevada and Cascade Mountains in Plumas County, California, USA (Fig 1). We sampled birds within 26 California Interagency Planning Watersheds that range in area from 1280–6324 ha [29]. Our study area was previously delineated by researchers conducting a multi-disciplinary study of the effects of fuel treatments on vertebrates, vegetation, and fire behavior [30]. The study area is comprised predominantly of Sierra mixed-conifer and true-fir cover types with smaller amounts of montane chaparral and hardwood-dominated habitat [31]. No specific permits were required to conduct this work either by the land manager or wildlife agency and the coordinates of all study locations are available for download at: http://data.prbo.org/apps/snamin/index.php?page=fuels-view-study-locations. This study did not involve any threatened or endangered species.

### Defining Spotted Owl management areas

Spotted Owl Home Range Core Areas (Core Areas) are a U.S. Forest Service land allocation of the best available 405 ha (1000 acres) of habitat associated with each known Spotted Owl nest-site or territory [11]. The best available owl habitat is defined as areas where two or more tree canopy layers are present, trees in the dominant crown class have an average diameter at breast height over 61 cm (24 inches), canopy cover exceeds 50%, and trees over 7.3 m (24 feet) crown diameter are present. Core Areas are protected from major anthropogenic disturbance that would significantly reduce canopy cover or otherwise alter habitat suitability for Spotted Owl. Core Areas are designated after the presence of a nesting or roosting owl is confirmed and they are retained administratively, unless specifically petitioned for removal following major habitat alteration (e.g. stand replacing fire). Since Spotted Owls had been intensively monitored across our study area since at least 2002 [30, 32], there were very few if any breeding owl territories that had not been detected and assigned Core Areas.

### Site selection

We established three to four point count transects, each consisting of 12 stations, in each of the 26 planning watersheds in the study area. For each transect, we used GIS [33] to randomly select a starting point within 500 m of a drivable road. We then added 11 additional point count stations along a random compass bearing from the starting point spaced at 250 m intervals. If transects could not be established using a random bearing due to inaccessible areas being encountered (e.g., private property, prohibitively steep topography), we chose a non-random bearing or placed stations adjacent to (> 50 m off) the nearest road. We established a total of 1092 stations along 91 transects in this manner. We added an additional 72 stations in clusters of two to four within Core Areas to increase our sample size of known Spotted Owl habitat. These 72 stations were established by selecting Core Areas adjacent to existing transects (to reduce effort to survey them), with confirmed nesting activity in the previous five years; when combined with the existing Core Area stations they represented a sample well distributed across the study area. We used existing digitized polygons of Core Areas to delineate each station location as either inside or outside Core Areas in ARC GIS 9.2 [34]. The total sample size was 1164 stations on 109 transects, with 608 outside and 556 inside of Core Areas. Survey stations ranged in elevation from 944 to 2140 m and encompassed considerable variation in topography, with slopes ranging from 0 to 65 degrees and a full range of aspects.

### Survey protocol

We used standardized five-minute multiple distance bin point count surveys [35–36] to sample the avian community. At each station, we recorded all birds detected (visual or auditory) and estimated their distance from the observer. We visited each station twice between May 15 and June 30 in both 2005 and 2006 for a detection history of four total visits. We completed counts within four hours of sunrise and did not survey during inclement weather. All observers completed intensive two-week training sessions and passed a double observer field test on bird identification, with an experienced crew supervisor, prior to conducting counts. No animal ethics committee was consulted prior to this study as we used a well-established passive survey of short duration that has minimal impact on study species, none of which are threatened or endangered.

At each station we characterized the vegetation within 50m of the survey plot center using visual estimates of the percent of the plot that was covered by each of overstory trees, shrubs, and herbaceous species and assigned habitat types to each station using California Wildlife Habitat Relationships [31]. We measured structural characteristics including basal area (a measure of woody cross-sectional area in standing trees) using a 10 factor key from the center of the station, diameter at breast height of the largest tree in the plot (measured with a tape), and the average height of canopy trees (estimated using a laser range finder). We also sampled elevation, aspect, and slope at each station center from the Sierra Nevada Regional Digital Elevation Model [37]. We summarized these data for all stations and compared average values between the inside vs. outside Core Area groups of stations using a T-test (2-tailed, unequal variance between groups).

### Analysis

We recorded a total of 90 species on these surveys but removed 9 species (waterfowl, nocturnal species, non-breeding migrants, unidentified species, and a hybrid) for which we felt our point count methodology generated an inappropriate sample [35]. The resultant list included 81 species of breeding landbirds. All 81 species were included in a hierarchical Bayesian multiple-species occupancy model. Counts were tallied for all individuals detected within 100m of the observer. Since we did not remove species due to rarity they ranged in abundance from Hermit Warbler (*Setophaga occidentalis*) with 5962 detections to Clark’s Nutcracker (*Nucifraga columbiana*) with only a single detection.

We followed closely the methods of Zipkin et al. [38], and conducted the analyses with the statistical package R version 3.0.1 x64 [39] utilizing the RJAGS [40] and R2JAGS [41] packages. We defined the occurrence of species i at site j as a Bernoulli random variable where occurrence = 1 and absence = 0, such that z(i,j) ~ Bern(ψ_i,j_) where ψ_i,j_ is the probability that species i occurs at site j. The detection model for species i at station j during visit k is defined as x(i,j,k) ~ Bern(p_i,j,k_) where p_i,j,k_ is the probability of detection of species i at station j on visit k given that the species is present. We visited each station exactly two times in each year and defined sampling units as station-year combinations, thus ‘stacking’ the yearly detection histories. This data format achieves a larger effective sample size while allowing for assumption of open populations at survey locations since occupancy in one year is independent of occupancy in the other year. This design makes it possible to fit a larger set of occupancy covariates for some less prevalent species and avoid problems associated with poor model fit, at the expense of potentially underestimating the error around parameter estimates.

We assumed that occurrence and probability of detection vary by species and are functions of habitat and survey covariates incorporated via the logit link function [42]. The probability of occurrence for species i at station j depended on whether station j was inside or outside a Core Area, year of survey, tree cover, shrub cover (not including tree seedlings or saplings), solar radiation index (SRI) [43], and linear and squared terms (to account for associations with intermediate values) for elevation. All continuous-scale covariates were standardized (mean = 0.0, standard devation = 1.0), and elevation was standardized prior to calculating the squared term. Thus the quadratic elevation term returns high values for both high and low elevations, and low values for elevations near the mean. None of the variables were highly correlated (all R<0.21). The probability of detection for species i at station j on visit k depended on whether the visit occurred in 2005 or 2006, the day of year and time of day of each visit, and the measured tree basal area at each station. Detection covariates were also standardized. Because basal area was correlated with both tree cover (R = 0.33) and shrub cover (R = 0.31), we only included basal area as a detection covariate. We selected basal area because we felt it was the most proximate vegetation feature influencing observers’ ability to hear bird vocalizations as well as visually detect individuals.

Occupancy models need information to resolve the uncertainty between non-detections and non-occurrences in repeated sampling data. We found large standard errors on occupancy estimates from preliminary models that did not include any habitat variables as covariates of occupancy. Thus, we included covariates such as tree and shrub cover to help inform the model for the many species that are tied to particular ranges of these broad habitat features. However, including these covariates in the models does influence the Core Area effect. Thus, the associations we found with Core Area are beyond any differences related to the basic habitat conditions of shrub and tree cover. We assume that the Core Area parameter accounts for the many remaining factors that differ inside vs. outside of core areas and that influence species distributions.

In this hierarchical model (S1 Text) we treated the species-specific occurrence and detection parameters and all the covariate parameters as random effects governed by community-level hyper-parameters as described in Zipkin et al. [38]. All occupancy and detection covariates as well as the probability of detection parameter were initiated with non-informative priors. To speed convergence, occupancy was initialized with naïve occupancy of each species, which we defined as the proportion of the 1164 stations with at least one detection across all four visits (see S1 Text). The monitored model parameter estimates included all occupancy and detection probability covariates, the Core Area effect parameter for each species, probabilities of detection for each species and survey year, and the estimated species richness at each station. Estimated species richness at each station is a derived parameter, while all others are latent or random effects. We did not augment the species list with an arbitrary number of additional “undetected” species with all-zero detection histories as the total number of species in the community was not a parameter of interest. Also, preliminary model simulations with augmented species converged on the actual number of detected species which led us to abandon the augmentation approach in favor of a simpler model with shorter computation times. The model was run for 50,000 iterations with a burn-in of 10,000 iterations and sample thin = 50. We ran seven separate model chains in a parallel processing arrangement and summarized posterior distributions using the functions “coef” and “confint” in the RJAGS package. Convergence was assessed by calculating the Gelman-Rubin convergence statistic and ensuring that a value of <1.1 was achieved for all model parameters [44].

To assess parameter estimates we plotted 90% credible intervals from each posterior distribution and considered those that do not include 0.0 as significant. We counted the number of species with significant Core Area effects and assigned them into Core Area-associated and Core Area-avoiding groups. We plotted model coefficient values for all species detected at 10 or more stations (at least one detection on 4 visits across 2 years, n = 54) and error bars representing 90% credible intervals. Credible intervals were very large for the remaining less prevalent species (n = 27), and so we limited our summary analyses to the 54 more prevalent species. To evaluate relative explanatory power of the occupancy covariates while accounting for the different variable ranges between the binary Core Area effect, standardized covariates, and the squared elevation term, we calculated a statistic approximating the chi-squared with large sample size using the squared value of the model coefficient divided by its standard error [45]. We then averaged these values across the 54 most prevalent species and ranked the covariates from largest to smallest as a relative measure of influence on occupancy across our entire avian community.

We highlighted the results for the 12 Partners in Flight conifer forest focal species [46] that occurred in our dataset. These species included: Black-backed Woodpecker (*Picoides arcticus*), Black-throated Gray Warbler (*Setophaga nigrescens*), Brown Creeper (*Certhia americana*), Dark-eyed Junco (*Junco hyemalis*), Fox Sparrow (*Passerella iliaca*), Golden-crowned Kinglet (*Regulus satrapa*), MacGillivray's Warbler (*Geothlypis tolmei*), Olive-sided Flycatcher (*Contopus cooperi*), Pileated Woodpecker (*Dryocopus pileatus*), Red-breasted Nuthatch (*Sitta candadensis*), Vaux's Swift (*Chaetura vauxi*), and Western Tanager (*Piranga ludoviciana*).

In addition to the Bayesian hierarchical model, we fit occupancy using the same model structure and covariates for all species with the “occu” function in the R package Unmarked [47]. We used these results to verify parameter estimates from the Bayesian model, and to assess the utility of the multi-species model for fitting effects for rare species that could not be analyzed with single species models because of sparse data.

We further investigated the potential Spotted Owl umbrella by assessing the conservation status of each species according to three different scoring systems; California Bird Species of Special Concern (BSSC) [13], Climate Vulnerability (CV) [48], and Regional Conservation Score—breeding season (RCS-b) from the Partners in Flight Species Assessment (S1 Table) [49]. We then compared the scores of species with significant associations with Core Areas using a T-test (2-tailed, unequal variance between groups). In addition to comparing the average scores, we counted the number of species in each group with the highest conservation concern scores (more than 0.5 standard deviation above the average scores of all 81 species) to evaluate if Core Areas are used preferentially by a larger proportion of species with the greatest conservation concern in our study area.

## Results

**Fig 2:**
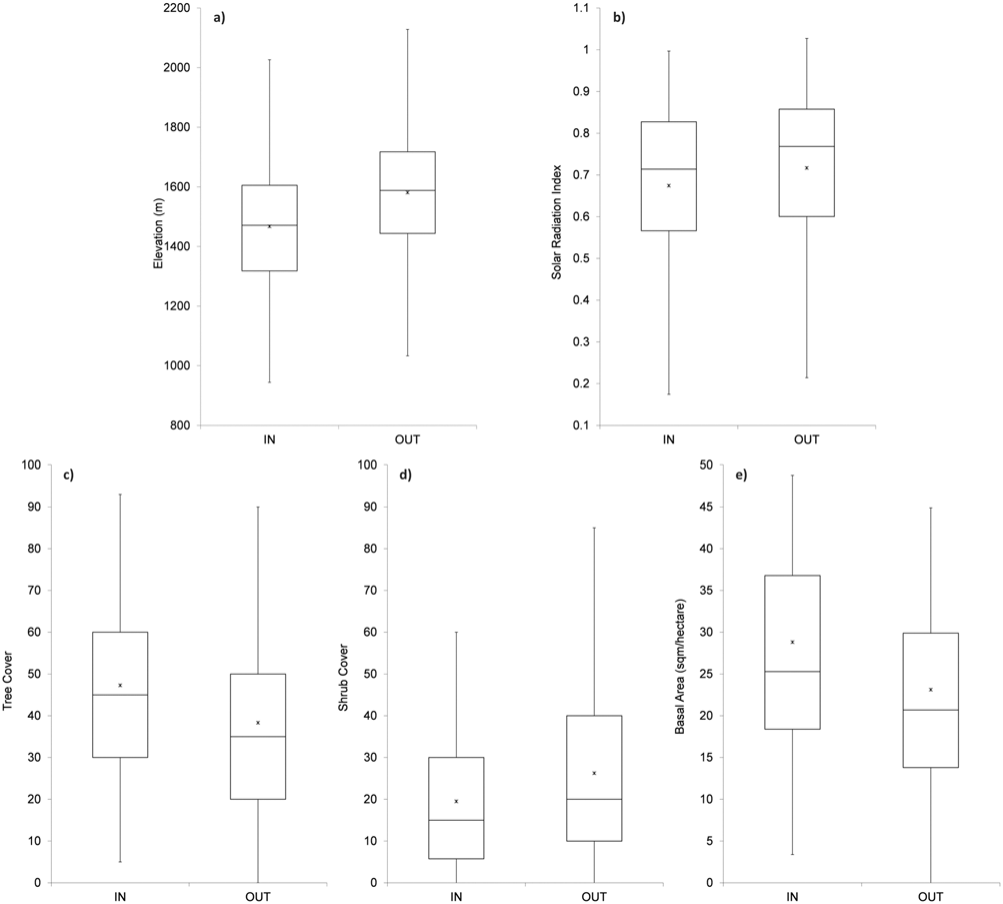
Field vegetation survey measurements and landscape topography variables within and outside of spotted owl Core Areas. All mean values are significantly different (p<0.01) using a two-tailed unequal variance t-test (n = 556 inside Cores, 608 outside). Box plots are shown listing the median, first and third quartiles, mean (asterisk), and error bars depict 1.5 * interquartile range. Subfigures include: A) elevation, B) Solar radiation index, C) tree cover, D) shrub cover, E) basal area.

Vegetation cover and structure was different inside of Core Areas than the surrounding landscape. Average tree cover, tree height, and basal area were all higher inside Core Areas (p<0.001, 2-tailed T-test), while shrub and herbaceous cover were higher outside Core Areas (Fig 2). Core Areas occurred at lower elevations and sites with less solar radiation on average. Though the survey transects are randomly distributed and frequently overlap Core Area boundaries, the group of point count stations inside the Core Areas had a higher proportion of dense mature forest even though the ranges of all measured variables largely overlap. The habitat types inside Core Areas were dominated by Sierra mixed-conifer (89%) and True-fir (*Abies magnifica* and *concolor*) forest (11%). Only one station inside Core Areas was classified as mixed hardwood-conifer, and two stations were classified as montane chaparral. Stations outside Core Areas were distributed more broadly among forest types with Sierra mixed-conifer (76%), Truefir (17%), chaparral (4%), mixed hardwood-conifer (2%), and ponderosa pine (*Pinus ponderosa*; 2%). Many of the forest habitat classes intergrade with chaparral (only 10% tree cover is required to characterize a site as forest rather than chaparral) both within and outside Core Areas, and thus these broad forest type classifications do not reflect finer scale factors that are certainly driving some of the variation in the avian community.

**Fig 3:**
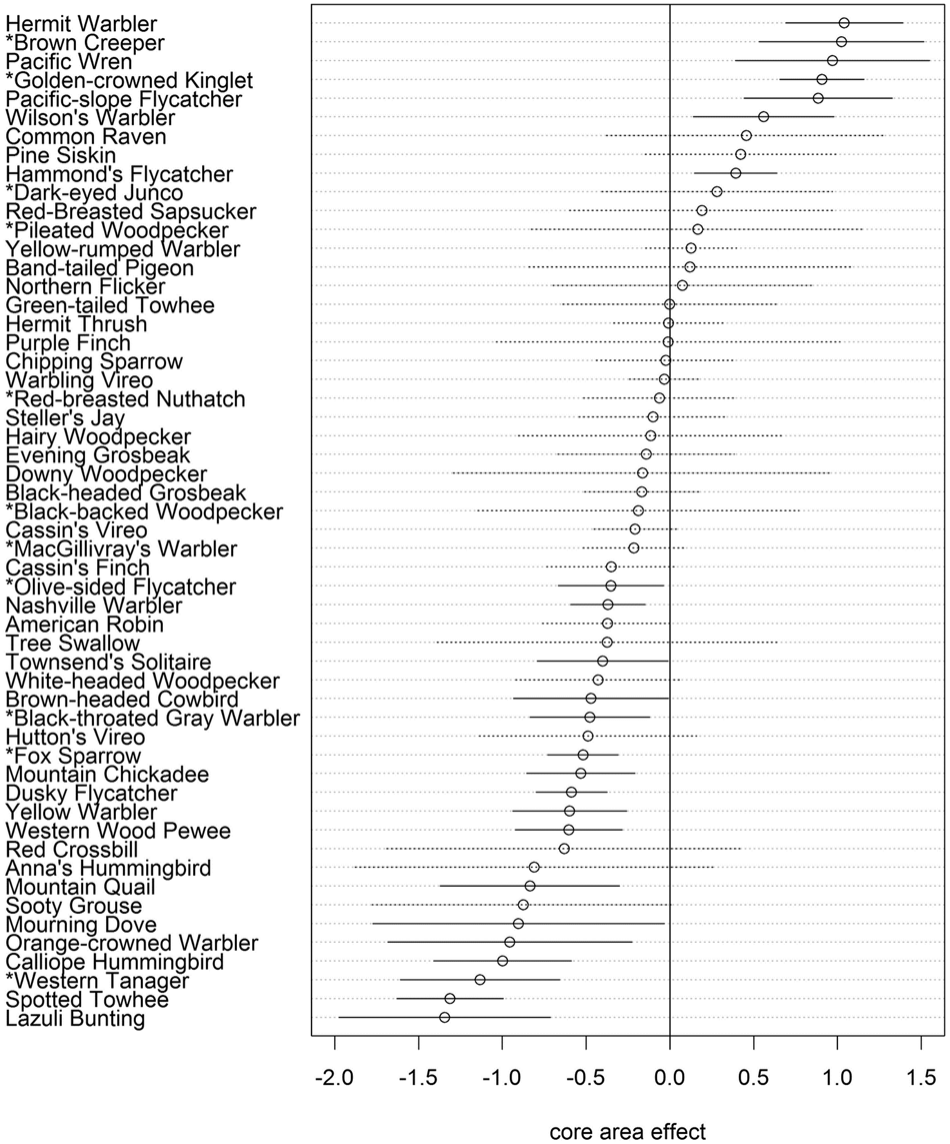
Core Area effects for 54 species detected at more than 10 point count stations. Error bars show 90% credible intervals. Species with significant Core Area effects are shown with solid lines, insignificant effects are shown with dotted lines. Partners in Flight conifer forest focal species are listed with an asterisk.

Seven species had a positive Core Area effect and 17 species had a negative Core Area effect (Fig 3). The estimated species richness per station was significantly higher outside Core Areas (23.1) than inside (21.7; p<0.001). Without considering statistical significance, 65 of the 81 species analyzed had negative Core Area effects while only 16 were positive. Many species had large (< -0.35) non-significant negative Core Area coefficients, and 26 out of 27 of the rarest species (detected at fewer than 10 stations) had negative Core Area coefficients but, due to small sample sizes these effects were not statistically significant.

The 12 Partners in Flight conifer forest focal species were distributed across all values of Core Area association, from the second highest value among all species (Brown Creeper, 1.02) to the third lowest (Western Tanager, -1.13), which suggests that they represent a broad range of habitat conditions (see S1 Table for species scientific names). They followed a similar pattern as the broader community with two species having positive Core Area effects (Brown Creeper and Golden-crowned Kinglet) and four negative (Olive-sided Flycatcher, Black-throated Gray Warbler, Fox Sparrow, and Western Tanager; Fig 3).

**Fig 4:**
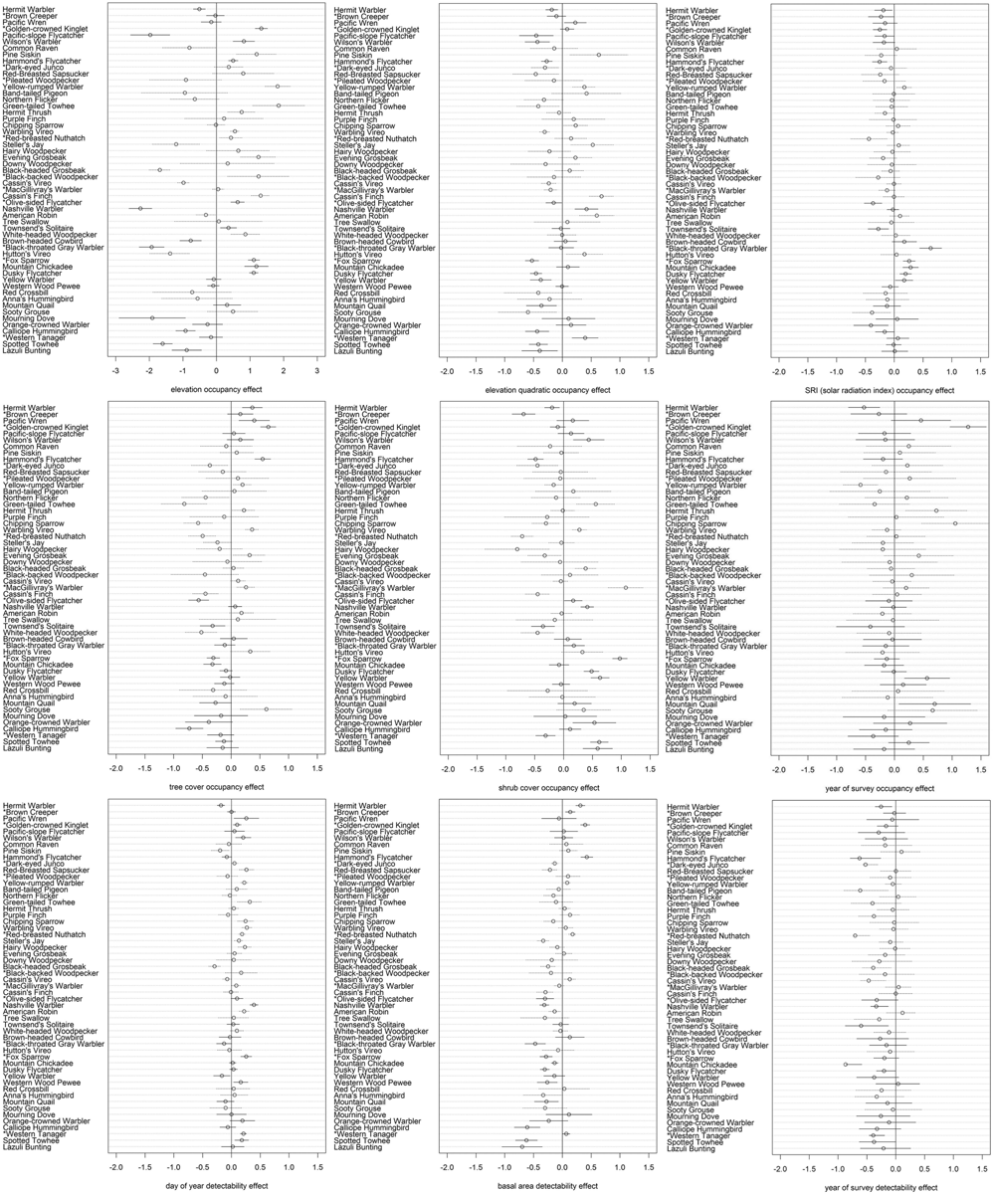
Occupancy model covariate effects for 54 species detected at more than 10 point count stations. Error bars show 90% credible intervals. Subfigures include: A) elevation (occupancy), B) elevation squared (occupancy), C) SRI (occupancy), D) year of survey (occupancy), E) tree cover (occupancy), F) shrub cover (occupancy), G) year of survey (detection), H) date of survey (detection), I) basal area (detection). Species with significant Core Area effects are shown with solid lines, insignificant effects are shown with dotted lines. Partners in Flight conifer forest focal species are listed with an asterisk.

Elevation effects were significant for 33 out of 54 species; with 18 favoring higher elevation and 15 lower (Fig 4). Elevation quadratic effects were significant for 29 species: 19 negative—indicating an association with an intermediate elevation, and 10 positive—indicating an association with elevations at one of the extremes within our study area. Solar radiation index was significant for 16 species: 6 positive—indicating an association with southern aspect, and 10 negative—indicating an association with northerly aspects (Fig 4).

Tree cover and shrub cover had effects on the occupancy of 21 and 23 species respectively. Tree cover was positive for 9 species, and negative for 12 species (Fig 4). Shrub cover was positive for 12 species, and negative for 11 species. Most Core Area associates were positively associated with tree cover (except Hammond’s Flycatcher), and the Core Area avoiding species were negative or not significant. The majority of Core Area avoiding species had positive shrub cover effects (with the exception of Western Tanager and Townsend’s Solitaire, Fig 4). Three of the seven Core Area associated species had negative shrub cover effects, while one (Wilson’s Warbler) was positive.

The most influential occupancy covariates on average across all 54 species were elevation (average χ^2^ approximation metric = 26.1), followed by shrub cover (11.1), elevation^2^ (7.2), tree cover (6.7), Core Area (5.6), SRI (3.2), and year (2.0).

The average probability of detection across all species was 0.20 in 2005 and 0.24 in 2006. The species with the highest probability of detection (averaged over two years) was Hermit Warbler (0.81). There were 36 species that had probability of detection less than 0.1, and these were largely the species detected at very few stations. The day of year detectability covariate was significant for 21 species, 17 of which were positive indicating higher detectability later in the survey season. Basal area was significant for 25 species, with 8 positive and 17 negative. The majority of Core Area avoiding species had a negative effect (Fig 4).

When we compared the results from our Bayesian multi-species occupancy model with those from single-species occupancy models for the 41 species that could be fitted using Unmarked, the Core Area effects for each species were very similar between the two models (R^2^ = 0.98). The remaining 40 species models did not converge in Unmarked. Since we include results from 54 of the 81 species included in the Bayesian multi-species model, this method allowed us to evaluate 13 additional species over the Unmarked methods, 6 of these species had a negative Core Area effect, and the remaining 7 showed no significant association. The credible (Bayes) and confidence (Unmarked) intervals were largely similar between models (R^2^ > 0.96).

Conservation indices showed some apparent differences between groups of species with different Core Area associations. The 7 Core Area associated species on average had higher (indicating greater risk) Bird Species of Special Concern (BSSC) scores than the 17 Core Area avoiding species (21.4 vs 12.2. P = 0.02). There was some evidence that Climate Vulnerability (CV) scores were higher for Core Area associated species (23.6 vs. 18.0, P = 0.09), but RCS-b was not different (14.0 vs. 13.4, P = 0.49). When we considered the number of species with relatively high conservation scores, there were as many or more species in the Core Area avoiding group for every metric (BSSC = 21 or higher: 2 outside vs. 2 inside; CV = 24 or higher: 10 outside vs. 5 inside; RCS-b = 15 or higher: 6 outside vs. 3 inside). Also, the two species currently on the BSSC list in our dataset, Olive-sided Flycatcher and Yellow Warbler, both had negative Core Area effects. There were no species in our dataset that are listed under the U.S. or California endangered species acts.

## Discussion

### Effects of Spotted Owl-focused management on the avian community

This analysis represents the first to quantitatively assess the conservation efficacy of a surrogate species by using detection-corrected multi-species occupancy, rather than broad brush community metrics that ignore individual species patterns (e.g. richness or diversity). Our results support the conclusions from other studies that the strategy of employing a single rare species as a surrogate for a broader community of organisms is limited, and best done with caution and empirical evidence [3,10,50]. While, Core Areas may be an effective tool for managing habitat for spotted owls [51], the umbrella afforded by habitat protected in spotted owl reserves only supports increased occupancy for a small number of species compared to the surrounding landscape. Our results support other findings illustrating the importance of non-old growth closed canopy forest for birds in western North American forests [52–54].

In interpreting our results it is important to better understand what Core Areas are. Core Areas are human constructs that are generally anchored around nest sites and may not align perfectly with spotted owl home ranges. They are also not necessarily representative of the range of conditions occupied by the owl prior to euro-American alteration of the ecosystem. Based on habitat associations of their favored prey [55–56], and persistence in burned areas [57–58], the species commonly uses open habitats for foraging, but these areas are not typically included in Core Areas, which are intended to protect nesting areas. Thus, our findings should be interpreted in the context of the current direction in Core Area design, and not necessarily the umbrella that would result from protecting entire owl home ranges.

An estimate of the population trends or vital rates of the species (e.g. survival, productivity) associated with Core Areas would provide more confidence that the patterns we detected reflect differences in habitat quality [59–60], and thus the ability or inability of Core or non-Core Areas to promote viable populations of their associated species. One might expect that areas more heavily influenced by non-natural treatments would act as ecological traps [61]. However, existing evidence suggests that in western forests the reproductive success of species associated with areas altered by silvicultural applications can be relatively high [62–63]. Our finding of higher occupancy for many species outside of Core Areas should be a reasonable proxy for habitat quality.

### Disturbance-dependent species

The majority of the species negatively associated with Core Areas are tied to shade-intolerant broad leaf trees, chaparral, and forest edges. Natural disturbance in ecological systems creates and maintains habitats used by a diverse assemblage of avian species [64] and the importance of early successional habitats for birds in western forests is well documented [52, 65–67]. Many of the species negatively associated with Core Areas, especially the shrub associates Lazuli Bunting, Mountain Quail, Yellow Warbler, Spotted Towhee, and Fox Sparrow, were far more abundant in portions of the landscape surrounding our study area that had burned in mixed severity fires in the past 2–12 years [68]. Species such as Nashville Warbler and Black-throated Gray Warbler are closely aligned with broadleaf trees, especially oak (*Quercus spp.*), that occur in more open canopy mixed conifer forest in the Sierra Nevada.

Disturbance in western North American forests is not only important for providing the full range of habitat types occupied by birds, but the juxtaposition of distinct habitat types [69–70], and within stand structural diversity [71–72] also influence patterns of avian diversity in temperate coniferous forest. The Olive-sided Flycatcher and Western Wood-Pewee are strongly associated with edges—where mature forests intersect openings [65–66,69,73]. Both of these species had a strong negative association with Core Areas and both are among the fastest declining landbirds in the Sierra Nevada [74]. Western Tanager and Calliope Hummingbird had among the strongest negative associations with Core Areas. Calliope Hummingbirds require nectar sources that primarily occur on shade intolerant understory plant species. In our study area, Western Tanager responded positively to moderate severity fire and fuel reduction treatments [68, 75]. Managing for shade intolerant broad leaf plant assemblages and increased open forest conditions outside of dense forest reserves in conifer dominated western forests appears fundamental to sustaining avian diversity [52,55,67].

The difference of 1.4 more species per point count station outside of Core Areas likely reflects the importance of forest heterogeneity in promoting avian diversity [52]. Though the difference in species richness was fairly small, the scale at which we measured it was also small. Thus, when extrapolated to our entire study area or larger, a conversion to Core Area like habitat could result in a substantial decline in avian richness within these forest stands. Understanding the drivers of avian diversity and the habitat associations and response to management of the species most negatively associated with Core Areas can provide guidance for managing outside of these reserves.

### Balancing Late-seral Reserves with Other Habitat Conditions

Our results suggest that promoting the expansion and densification of late-seral closed canopy forest in the Sierra Nevada should be done with a measured approach. In our study area 32% of the National Forest land was in a designated Core Area. When other designations that promote similar conditions (e.g. Wilderness, Northern Goshawk [*Accipiter gentilis*] reserves, riparian conservation areas), and logistical restrictions (e.g. steep terrain, road less areas) are considered, a substantial portion of the study area occurs where mechanical treatments that may mimic natural disturbance are restricted. Additionally, a pervasive fire suppression policy has dramatically reduced the area burning annually over at least the last seven decades [20–21]. Dense conifer forest may now occur in many places where it did not prior to euro-American influence [26,76]. That the majority of the rarest species in our study area had some evidence of a negative association with Core Areas suggests that forest densification may have already altered avian community composition in the Sierra Nevada.

With a changing climate the patterns and processes that shape the Sierra Nevada—especially disturbance regimes—may result in unpredictable changes in forest structure and composition [77]. For example, fire extent and severity both appear to be increasing across the region in recent decades [78], though the area burning annually is still below levels from previous centuries [20–21]. Regardless of the future trends in fire, fire severity, and forest densification, with the importance of non-closed canopy forests in maintaining bio-diversity, land managers of western forests should proactively manage for these habitat conditions and not assume that changes in climate and fire patterns will create sufficient quantities in the appropriate locations and spatial patterns required by wildlife. In order to help ensure the needs of all species are being met, especially on the lands not being managed for Spotted Owl and other late seral species, balancing a Core Area approach with one that promotes habitat for the majority of the avian community in the surrounding landscape appears prudent to sustaining avian diversity in fire prone western forests.

### Evaluation of Modeling Approach

The Bayesian modeling approach allowed us to include many uncommon species in the analysis, but we still chose not to evaluate estimates for the 27 rarest species in our dataset due to the low confidence in parameter values. We feel that it was beneficial to initially include all of these species in the model as it allowed us an unbiased assessment of the entire avian community rather than selecting an arbitrary, and potentially biased, set of focal species. This method has also been shown to provide an accurate assessment of species richness [42], which would not have been possible with single species occupancy models. The results from both the Bayesian and single-species methods were very similar for the species for which models converged, including very similar confidence intervals for parameter estimates. But using results from the Bayesian approach allowed us to evaluate more species, and reinforced the conclusion that a large proportion of the avian community is not protected under the Spotted Owl Core Area umbrella.

To obtain well-fitted models with meaningful confidence intervals for a large number of species, it was necessary to include vegetation covariates. We attempted to include the smallest number of covariates necessary to inform the model by accounting for broad habitat conditions at the point count stations even though these conditions were shown to be significantly different inside vs. outside Core Areas. For some species, the tree cover and shrub cover variables account for some of the variation in their occurrence that could be attributable to the Core Area effect and thus dampen their overall Core Area association (e.g. Fox Sparrow). Some species showed significant Core Area effects despite having associations with shrub cover that were opposite of the expected direction. For example, Wilson’s Warbler had positive Core Area and shrub cover effects, and Western Tanager and Townsend’s Solitaire had negative Core Area and shrub cover effects. The Core Area parameter accounts for a potentially very large number of factors that influence species occurrence, including: landscape patterns; disturbance history; soil productivity; micro climate; and numerous other vegetation composition and structural conditions that vary between Core and non-Core Areas. These differences in landscape, soil, climate, and vegetation patterns presumably vary at fine scales (roughly 50–500m radius) since the majority of transects contained stations both within and outside Core Areas. This would result in landscape patterns measured at coarser scales of >1km resolutions being very similar between the inside and outside Core Area groups. As it was outside of the scope of this paper to evaluate each of these factors individually, we chose instead to look for very general differences in the avian community inside vs. outside the Core Areas by including it as a single parameter in the model. This small set of covariates seems to optimize the balance between model quality and assessment of Core Area effects after accounting for these very general habitat conditions.

## Conclusion

In naturally heterogeneous ecosystems, single, habitat specialist species should be employed as surrogates with caution, preferably as one part of a multi-species monitoring approach. In the Sierra Nevada, current management emphasizing Spotted Owl and other late seral associated species, coupled with fire suppression, have the potential to be too broadly applied. As a result, the loss of forest heterogeneity (fine and landscape scale) and fire-dependent habitats may be a significant threat to avian diversity. With the loss of old growth forests, a condition shared among many forest ecosystems, it is clearly important to manage for late seral habitat. But, it is also essential to strike a balance with the needs of other organisms that are dependent upon other conditions. A more balanced approach that enhances these other habitat conditions is advisable to ensure biodiversity is sustained. As part of this program, we suggest a robust monitoring plan for a suite of avian species be employed as an effective surrogate for ensuring the needs of a greater number of species are being met across this and other large, heterogeneous ecosystems.

## Supporting Information

S1 Table Species scientific names and conservation scores. BSSC = California Bird Species of Special Concern, BSCV = California Bird Species of Climate Vulnerability, and RCS-b = Partners in Flight Regional Conservation Score—breeding season. Naïve occupancy is the proportion of sampling locations where the species was detected at least once over the four visits of this study.
(DOCX)

S1 Text JAGS model code. (DOCX)
