# Supplementary material for: A Quantitative Evaluation of the Conservation Umbrella of Spotted Owl Management Areas in the Sierra Nevada
Source: PLoS One. 2015 Apr 23;10(4):e0123778. doi: 10.1371/journal.pone.0123778 (PMC4408092; doi:10.1371/journal.pone.0123778)
Supplement: S1 Table — BSSC = California Bird Species of Special Concern, BSCV = California Bird Species of Climate Vulnerability, and RCS-b = Partners in Flight Regional Conservation Score—breeding season. Naïve occupancy is the proportion of sampling locations where the species was detected at least once over the four visits of this study. (DOCX) [file pone.0123778.s001.docx]

| Common Name | Scientific Name | BSSC | BSCV | RCS-b | Naïve Occupancy |
| --- | --- | --- | --- | --- | --- |
| Acorn Woodpecker | *Melanerpes formicivorus* | 0 | 0 | 13 | 0.003 |
| American Kestrel | *Falco sparverius* | 10 | 12 | 11 | 0.002 |
| American Robin | *Turdus migratorius* | 12.5 | 20 | 11 | 0.331 |
| Anna's Hummingbird | *Calypte anna* | 0 | 0 | 12 | 0.021 |
| Band-tailed Pigeon | *Patagioenas fasciata* | 27.5 | 16 | 17 | 0.055 |
| Bewick's Wren | *Thryomanes bewickii* | 7.5 | 18 | 10 | 0.001 |
| Black-backed Woodpecker | *Picoides arcticus* | 17.5 | 35 | 13 | 0.011 |
| Black-headed Grosbeak | *Pheucticus melanocephalus* | 15 | 24 | 12 | 0.268 |
| Black-throated Gray Warbler | *Setophaga nigrescens* | 5 | 24 | 14 | 0.149 |
| Blue-gray Gnatcatcher | *Polioptila caerulea* | 27.5 | 18 | 9 | 0.002 |
| Brown Creeper | *Certhia americana* | 20 | 20 | 15 | 0.459 |
| Brown-headed Cowbird | *Molothrus ater* | 0 | 0 | 11 | 0.062 |
| Calliope Hummingbird | *Selasphorus calliope* | 17.5 | 28 | 15 | 0.116 |
| Cassin's Finch | *Haemorhous cassinii* | 17.5 | 20 | 18 | 0.150 |
| Cassin's Vireo | *Vireo cassinii* | 27.5 | 24 | 15 | 0.470 |
| Cedar Waxwing | *Bombycilla cedrorum* | 0 | 25 | 7 | 0.002 |
| Chestnut-backed Chickadee | *Poecile rufescens* | 25 | 24 | 12 | 0.006 |
| Chipping Sparrow | *Spizella passerina* | 27.5 | 20 | 13 | 0.131 |
| Clark's Nutcracker | *Nucifraga columbiana* | 15 | 20 | 16 | 0.001 |
| Common Raven | *Corvus corax* | 0 | 0 | 8 | 0.030 |
| Cooper's Hawk | *Accipiter cooperii* | 22.5 | 15 | 14 | 0.003 |
| Dark-eyed Junco | *Junco hyemalis* | 20 | 20 | 12 | 0.852 |
| Downy Woodpecker | *Picoides pubescens* | 20 | 15 | 10 | 0.009 |
| Dusky Flycatcher | *Empidonax oberholseri* | 7.5 | 24 | 14 | 0.622 |
| Evening Grosbeak | *Coccothraustes vespertinus* | 20 | 25 | 15 | 0.130 |
| Fox Sparrow | *Passerella iliaca* | 17.5 | 30 | 11 | 0.373 |
| Golden-crowned Kinglet | *Regulus satrapa* | 15 | 25 | 15 | 0.582 |
| Gray Jay | *Perisoreus canadensis* | 25 | 30 | 9 | 0.004 |
| Green-tailed Towhee | *Pipilo chlorurus* | 0 | 0 | 15 | 0.036 |
| Hairy Woodpecker | *Picoides villosus* | 20 | 20 | 12 | 0.164 |
| Hammond's Flycatcher | *Empidonax hammondii* | 17.5 | 24 | 14 | 0.345 |
| Hermit Thrush | *Catharus guttatus* | 7.5 | 25 | 12 | 0.361 |
| Hermit Warbler | *Setophaga occidentalis* | 30 | 28 | 16 | 0.912 |
| House Wren | *Troglodytes aedon* | 0 | 0 | 11 | 0.003 |
| Hutton's Vireo | *Vireo huttoni* | 10 | 16 | 15 | 0.046 |
| Lazuli Bunting | *Passerina amoena* | 20 | 18 | 14 | 0.043 |
| Lesser Goldfinch | *Spinus psaltria* | 12.5 | 12 | 12 | 0.002 |
| Lincoln's Sparrow | *Melospiza lincolnii* | 20 | 35 | 9 | 0.006 |
| MacGillivray's Warbler | *Geothlypis tolmiei* | 12.5 | 28 | 14 | 0.405 |
| Mountain Bluebird | *Sialia currucoides* | 0 | 0 | 12 | 0.002 |
| Mountain Chickadee | *Poecile gambeli* | 12.5 | 16 | 15 | 0.811 |
| Mountain Quail | *Oreortyx pictus* | 5 | 30 | 17 | 0.091 |
| Mourning Dove | *Zenaida macroura* | 5 | 12 | 6 | 0.015 |
| Nashville Warbler | *Oreothlypis ruficapilla* | 17.5 | 24 | 16 | 0.649 |
| Northern Flicker | *Colaptes auratus* | 17.5 | 16 | 14 | 0.122 |
| Northern Goshawk | *Accipiter gentilis* | 37.5 | 20 | 15 | 0.004 |
| Olive-sided Flycatcher | *Contopus cooperi* | 35 | 24 | 17 | 0.208 |
| Orange-crowned Warbler | *Oreothlypis celata* | 0 | 0 | 11 | 0.028 |
| Osprey | *Pandion haliaetus* | 22.5 | 35 | 11 | 0.002 |
| Pacific Wren | *Troglodytes pacificus* | 17.5 | 16 | 13 | 0.040 |
| Pacific-slope Flycatcher | *Empidonax difficilis* | 17.5 | 24 | 13 | 0.074 |
| Pileated Woodpecker | *Dryocopus pileatus* | 22.5 | 30 | 14 | 0.049 |
| Pine Siskin | *Spinus pinus* | 20 | 20 | 11 | 0.100 |
| Purple Finch | *Haemorhous purpureus* | 15 | 16 | 16 | 0.067 |
| Red Crossbill | *Loxia curvirostra* | 0 | 30 | 11 | 0.009 |
| Red-breasted Nuthatch | *Sitta canadensis* | 0 | 0 | 12 | 0.790 |
| Red-breasted Sapsucker | *Sphyrapicus ruber* | 22.5 | 20 | 17 | 0.157 |
| Red-tailed Hawk | *Buteo jamaicensis* | 0 | 0 | 10 | 0.006 |
| Red-winged Blackbird | *Agelaius phoeniceus* | 0 | 0 | 8 | 0.003 |
| Ruby-crowned Kinglet | *Regulus calendula* | 0 | 0 | 9 | 0.003 |
| Sharp-shinned Hawk | *Accipiter striatus* | 22.5 | 20 | 13 | 0.003 |
| Song Sparrow | *Melospiza melodia* | 0 | 0 | 8 | 0.006 |
| Sooty Grouse | *Dendragapus fuliginosus* | 22.5 | 30 | 14 | 0.011 |
| Spotted Towhee | *Pipilo maculatus* | 0 | 0 | 13 | 0.170 |
| Steller's Jay | *Cyanocitta stelleri* | 5 | 24 | 16 | 0.389 |
| Swainson's Thrush | *Catharus ustulatus* | 32.5 | 30 | 10 | 0.004 |
| Townsend's Solitaire | *Myadestes townsendi* | 20 | 24 | 15 | 0.193 |
| Tree Swallow | *Tachycineta bicolor* | 27.5 | 24 | 10 | 0.011 |
| Turkey Vulture | *Cathartes aura* | 10 | 15 | 8 | 0.002 |
| Vaux's Swift | *Chaetura vauxi* | 37.5 | 35 | 14 | 0.003 |
| Warbling Vireo | *Vireo gilvus* | 35 | 24 | 12 | 0.353 |
| Western Bluebird | *Sialia mexicana* | 22.5 | 15 | 10 | 0.001 |
| Western Tanager | *Piranga ludoviciana* | 0 | 0 | 13 | 0.859 |
| Western Wood-Pewee | *Contopus sordidulus* | 12.5 | 24 | 14 | 0.141 |
| White-breasted Nuthatch | *Sitta carolinensis* | 0 | 0 | 11 | 0.004 |
| White-headed Woodpecker | *Picoides albolarvatus* | 17.5 | 20 | 16 | 0.180 |
| Williamson's Sapsucker | *Sphyrapicus thyroideus* | 10 | 24 | 18 | 0.002 |
| Wilson's Warbler | *Cardellina pusilla* | 32.5 | 28 | 12 | 0.114 |
| Wrentit | *Chamaea fasciata* | 30 | 28 | 18 | 0.003 |
| Yellow Warbler | *Setophaga petechia* | 32.5 | 28 | 12 | 0.125 |
| Yellow-rumped Warbler | *Setophaga coronata* | 0 | 0 | 11 | 0.791 |
