# Supplementary material for: A Quantitative Evaluation of the Conservation Umbrella of Spotted Owl Management Areas in the Sierra Nevada
Source: PLoS One. 2015 Apr 23;10(4):e0123778. doi: 10.1371/journal.pone.0123778 (PMC4408092; doi:10.1371/journal.pone.0123778)
Supplement: S1 Text — (DOCX) [file pone.0123778.s002.docx]

modF.fun <- function(){

int.mean ~ dunif(0,1)

mu.uint <- log(int.mean) - log(1-int.mean)

det ~ dunif(0,1)

mu.v <- log(det) - log(1-det)

mu.core ~ dunif(-10,10)

mu.2006 ~ dunif(-10,10)

mua1 ~ dunif(-10,10)

mua2 ~ dunif(-10,10)

mua3 ~ dunif(-10,10)

mua4 ~ dunif(-10,10)

mua5 ~ dunif(-10,10)

mub1 ~ dunif(-10,10)

mub2 ~ dunif(-10,10)

mu.2006 ~ dunif(-10,10)

sigma.uint ~ dunif(0,10)

sigma.v ~ dunif(0,10)

sigma.core ~ dunif(0,10)

sigma.2006 ~ dunif(0,10)

sigma.a1 ~ dunif(0,10)

sigma.a2 ~ dunif(0,10)

sigma.a3 ~ dunif(0,10)

sigma.a4 ~ dunif(0,10)

sigma.a5 ~ dunif(0,10)

sigma.b1 ~ dunif(0,10)

sigma.b2 ~ dunif(0,10)

sigma.2006 ~ dunif(0,10)

tau.uint <- 1/(sigma.uint*sigma.uint)

tau.v <- 1/(sigma.v*sigma.v)

tau.core <- 1/(sigma.core*sigma.core)

tau.2006 <- 1/(sigma.2006*sigma.2006)

tau.a1 <- 1/(sigma.a1*sigma.a1)

tau.a2 <- 1/(sigma.a2*sigma.a2)

tau.a3 <- 1/(sigma.a3*sigma.a3)

tau.a4 <- 1/(sigma.a4*sigma.a4)

tau.a5 <- 1/(sigma.a5*sigma.a5)

tau.b1 <- 1/(sigma.b1*sigma.b1)

tau.b2 <- 1/(sigma.b2*sigma.b2)

tau.2006 <- 1/(sigma.2006*sigma.2006)

for (i in 1:(n)) {

#Create priors for species i from the community level prior distributions

u.core[i] ~ dnorm(mu.core, tau.core)#I(-21,21)

u.2006[i] ~ dnorm(mu.2006, tau.2006)#I(-21,21)

u.int[i] ~ dnorm(mu.uint, tau.uint)#I(-21,21)

v[i] ~ dnorm(mu.v, tau.v)#I(-21,21)

a1[i] ~ dnorm(mua1, tau.a1)#I(-21,21)

a2[i] ~ dnorm(mua2, tau.a2)#I(-21,21)

a3[i] ~ dnorm(mua3, tau.a3)#I(-21,21)

a4[i] ~ dnorm(mua4, tau.a4)#I(-21,21)

a5[i] ~ dnorm(mua5, tau.a5)#I(-21,21)

b1[i] ~ dnorm(mub1, tau.b1)#I(-21,21)

b2[i] ~ dnorm(mub2, tau.b2)#I(-21,21)

det2006[i] ~ dnorm(mu.2006, tau.2006)

#Create a loop to estimate the Z matrix (true occurrence for species i at station j).

for (j in 1:J) {

logit(psi[j,i]) <- u.int[i] + u.core[i]*Pac[j] + u.2006[i]*Y2006[j] + a1[i]*elev1[j] +

a2[i]*elev2[j] + a3[i]*SRI1[j] + a4[i]*rlshrb1[j] + a5[i]*treecov1[j]

Z[j,i] ~ dbern(min(0.999, max(0.001, psi[j,i])))

#Create a loop to estimate detection for species i at station j during sampling period k.

for (k in 1:K[j]) {

logit(p[j,k,i]) <- v[i] + b1[i]*date1[j,k] + b2[i]*totba1[j] + det2006[i]*det.yr[j]

mu.p[j,k,i] <- p[j,k,i]*Z[j,i]

X[j,k,i] ~ dbern(min(0.999, max(mu.p[j,k,i], 0.001)))

}}}

#Create a loop to determine station level richness estimates

for(j in 1:J){

Nsite[j]<- sum(Z[j,])

}

}

#-----------------------------------------------#

# Run the model as a cluster (using rjags)

#-----------------------------------------------#

# load necessary libraries

library(dclone); library(snow); library(rjags); library(R2jags)

# MCMC settings

n.adapt <- 10000

n.update <- 10000

n.iter <- 50000

n.thin <- 50

n.chains <- 7

# create the cluster

cl <- makeCluster(7, type = "SOCK")

# create initialization that ensures independence of chains

inits2 <- jags.fit(sp.data, sp.params, modF.fun, n.chains = n.chains, n.adapt = 0, n.update = 0, n.iter = 0)$state(internal = TRUE)

# load the dclone package and set working directory for each cluster

clusterEvalQ(cl, library(dclone))

clusterEvalQ(cl, setwd('dir'))

# create list of data

cldata <- list(data=sp.data, params=sp.params, modF.fun, inits=inits2)

clusterExport(cl, "cldata")

# run the jags model on all clusters

jpfit <- jags.parfit(cl, data = sp.data, params = sp.params, modF.fun, inits = inits2, n.adapt = 0, n.update = n.update, n.iter = n.iter, thin = n.thin, n.chains = n.chains, progress.bar = "text")

stopCluster(cl)

jpfitvar<-varnames(jpfit, allow.null=TRUE)

jpfit.iter<-niter(jpfit)

jpfit.coef<-cbind(coef(jpfit),dcsd(jpfit),confint(jpfit, level=0.9))

colnames(jpfit.coef) <- c("coef","dcsd","10%","90%")
